# The Relationship between Subclinical Hypothyroidism and Carotid Intima-Media Thickness as a Potential Marker of Cardiovascular Risk: A Systematic Review and a Meta-Analysis

**DOI:** 10.3390/jcdd11040098

**Published:** 2024-03-25

**Authors:** Oana-Maria Isailă, Victor Eduard Stoian, Iuliu Fulga, Alin-Ionut Piraianu, Sorin Hostiuc

**Affiliations:** 1 Department of Legal Medicine and Bioethics, Faculty of Dentistry, “Carol Davila” University of Medicine and Pharmacy, 020021 Bucharest, Romania; sorin.hostiuc@umfcd.ro; 2 Department of Legal Medicine, Legal Medicine Service Dâmbovița, 130083 Târgoviște, Romania; 3 Department of Legal Medicine, Dunărea de Jos University, 800201 Galați, Romaniaalin.piraianu@gmail.com (A.-I.P.)

**Keywords:** carotid intima-media thickness, subclinical hypothyroidism, cardiovascular risk, screening

## Abstract

Background and Objectives: Thyroid dysfunction is known to have significant consequences on the cardiovascular system. The correlation between carotid intima-media thickness (CIMT) and subclinical hypothyroidism (SCH) has been frequently evaluated in clinical studies in recent years. This study aimed to evaluate the significance of this association through a meta-analysis. Methods: We conducted a systematic search of PubMed, MedLine, Scopus, and Web of Science databases using the keywords ‘subclinical hypothyroidism and carotid intima-media thickness’, from the beginning of each database until January 2023. We established the inclusion and exclusion criteria and considered studies that met the inclusion criteria. We used Jamovi for statistical analysis of the data. Results: We identified 39 observational studies that met the inclusion criteria, with 3430 subjects: 1545 SCH and 1885 EU. Compared to euthyroid subjects (EU), subjects with subclinical hypothyroidism (SCH) had significantly increased carotid intima-media thickness (CIMT) values; the estimated average mean difference was 0.08 (95% CI 0.05 to 0.10), *p* < 0.01, I^2^ = 93.82%. After the sensitivity analysis, a total of 19 from the 39 abovementioned studies were analyzed, with most studies showing a positive association between SCH and thickening of the carotid wall; the estimated average mean difference was 0.04 (95% CI 0.02 to 0.07), *p* = 0.03, I^2^ = 77.7. In addition, female sex, advanced age, and high cholesterol levels statistically significantly influenced this association. Conclusions: Our meta-analysis indicates a significant positive association between SCH and increased CIMT, but with some limitations.

## 1. Introduction

Subclinical hypothyroidism (SCH) is characterized by elevated serum thyroid-stimulating hormone (TSH) levels (above normal) and normal FT4 levels [1]. In most cases, this biohumoral alteration is insidious and is usually discovered incidentally, leading to a high variability of the reported prevalence, from 5.6% to 20.42%, depending on the population being subjected to analysis [2,3,4,5].

Carotid intima-media thickness (CIMT) is a measure of the thickness of the innermost layers of the carotid artery walls. It is used to determine the early stages of subclinical atherosclerosis, a condition in which the arteries become narrowed and hardened due to the buildup of plaque. CIMT is measured using ultrasound technology to analyze the thickness of the intima and the mean wall of the carotid artery. The relationship between CIMT and cardiovascular disease has been established, indicating the importance of this analysis. The factors responsible for atherosclerosis can lead to an increase in CIMT through hypertrophy of the intimal or medial carotid layers [6]. The use of CIMT in medical practice provides a noninvasive, reproducible, and cost-effective analysis with minimal risk to patients [7].

Multiple studies have shown an association between SCH and atherosclerotic cardiovascular disease. One of the early studies in this area was conducted by Hak et al. in the Rotterdam study to show a connection between subclinical hypothyroidism, aortic atherosclerosis, and myocardial pathology in elderly women with an autoimmune thyroid component. In addition, the study found that thyroid autoimmunity itself was not linked to carotid atherosclerosis or myocardial infarction [8]. In the Whickham survey, during 20 years of follow-up, a positive association was revealed between mortality following myocardial ischemic pathology and subclinical hypothyroidism [9].

Based on these findings, numerous recent studies have analyzed the association between SCH and increased CIMT as a cardiovascular risk factor, and the results have sometimes been discordant. The underlying mechanism of the positive association between subclinical hypothyroidism and vascular atheromatosis consists of a predisposition to endothelial dysfunction caused by TSH level by decreasing the endothelial response to vascular stimuli [10]; increasing inflammation and oxidative stress [11]; and through total cholesterol, triglycerides, and LDL cholesterol [12,13].

The purpose of this meta-analysis was to perform an updated analysis of the correlation between SCH and CIMT as a cardiovascular risk marker: an easy, noninvasive, and accessible marker that could constitute a good screening method for the prevention of cardiovascular pathology.

## 2. Materials and Methods

We undertook a study in adherence with the PRISMA guidelines for reporting systematic literature reviews and meta-analyses of observational studies [14].

### 2.1. Search Method

We conducted a systematized search in the PubMed, MedLine, Scopus, and Web of Science databases using the following keywords: “subclinical hypothyroidism and carotid intima-media thickness”, from the beginning of each database until January 2023. The baseline list of each study was reviewed for inclusion in the meta-analysis. We imported the references and summaries into the Mendeley Desktop software v1.19.8.

### 2.2. Selection Criteria

Inclusion criteria: Studies meeting the following inclusion criteria were included: (1) studies that analyzed the association between subclinical hypothyroidism and CIMT or from which this association could be investigated; (2) case-control studies; (3) persons in the control group had normal thyroid function with TSH values within the normal reference range; (4) CIMT value reported both for persons in the study group, with subclinical hypothyroidism, and for persons in the control group, with thyroid function in the normal reference range; (5) studies that also reported T4 value; (6) studies that reported 95% confidence interval.

In the case of studies analyzing the effectiveness of levothyroxine therapy, we used only the data presented for the subjects before initiating this treatment.

The following were excluded: (1) studies that did not provide any relevant information to obtain the necessary data; (2) series of cases/case presentations; (3) studies that analyzed persons with overt hypothyroidism or hyperthyroidism; (4) studies involving persons already undergoing treatment, reporting only drug therapy values; (5) non-control studies, animal studies, reviews; (6) studies that did not provide the mean value, standard deviation, or median parameters of interest.

### 2.3. Data Collection and Analysis

For each study, we conducted database research, extracted the data, and included it in Excel datasheets. The following information was obtained: author names, year, geographic region, TSH cut-off value, number of subjects, mean age, sex, BMI, TSH, CIMT, and lipidic profile.

In the case of studies that showed the mean value of the left CIMT and the mean value of the right CIMT separately, we calculated the common mean value by applying the formula for combined groups:

mean of total group = (n1 × X1 + n2 × X2)/(n1 + n2)

variance of total group = n1 × (S1^2^ + d1^2^) + n2 × (S2^2^ + d2^2^)/(n1 + n2)

where n1 = No. of observations in ‘region 1’, n2 = No. of observations in ‘region 2’, X1 = mean of region 1, X2 = mean of region 2, S1^2^ = variance of region 1, S2^2^ = variance of region 2 [15].

For studies reporting the median, we calculated the mean value based on the estimation method proposed by Luo et al. [16] and the standard deviation using the method proposed by Wan et al. [17,18].

For the studies that did not calculate the body mass index (BMI) using the classic method, the reported BMI value was not taken into account, being discordant with those from the rest of the included studies.

### 2.4. Quality Assessment

The methodological quality of each study was assessed according to the Newcastle–Ottawa scale (NOS) [19]. The scoring system consisted of three sections (case selection, comparability, and exposure) and the assessment included scores from 0 to 8 (Table 1). The closer the NOS score was to 8, the more methodologically qualitative the study was.

### 2.5. Statistical Analysis and Risk of Bias

Statistical analysis of data was carried out using Jamovi 2.3.21. The differences between the study and control groups were rendered as standardized mean difference (SMD) and 95% confidence interval for continuous-type variables. Differences were considered statistically significant at *p* < 0.05.

For the age difference mediator, we applied the formula

Ls_age_mean − Lc_age_mean

For the sex difference mediator, we applied the formula

(Ls_female/(Ls_female + Ls_male) − Lc_female/(Lc_female + Lc_male)) × 100

For the body mass index (BMI) moderator, we applied the formula

Ls_BMI_mean − Lc_BMI_mean

For the Cholesterol moderator, we applied the formula

Ls_Cho_mean − Lc_Cho_mean

For the LDL moderator, we applied the formula

Ls_LDL_mean − Lc_LDL_mean

For the HDL moderator, we applied the formula

Ls_HDL_mean − Lc_HDL_mean

For the Triglycerides moderator, we applied the formula

Ls_Tryglicerides_mean − Lc_Tryglicerides_mean

where Ls = study group, Lc = control group.

The analysis was performed using the mean difference as the outcome measure. A random effects model was used to fit the data. The amount of heterogeneity (tau^2^) was estimated using the DerSimonian–Laird estimator [59]. In addition, the Q-test of heterogeneity [60] and I^2^ statistics were analyzed, where I^2^ > 50% indicated significant heterogeneity and I^2^ < 25% was most likely not significant heterogeneity.

For publication bias analysis, we visually analyzed the symmetry of the funnel diagram, the rank correlation test, and the regression test using the standard error of the observed outcomes as predictors.

## 3. Results

### 3.1. Study Selection

**Figure 1:**
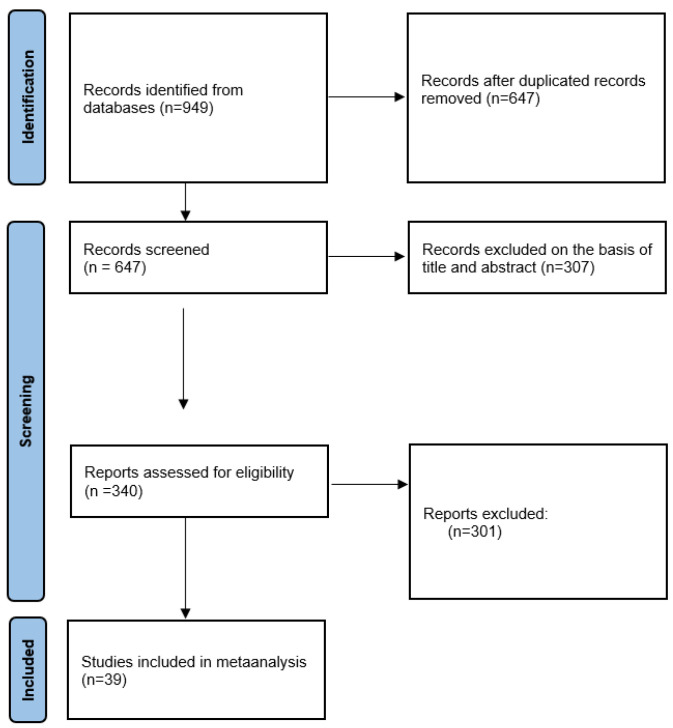
Search synthesis. Prisma flow diagram [14].

Following the initial analysis of the database, we obtained 949 articles. After excluding duplicates and irrelevant studies, we finally included 39 studies for further evaluation. (Figure 1, Table 2, Table 3 and Table 4).

### 3.2. CIMT in SCH Versus EU

**Figure 2:**
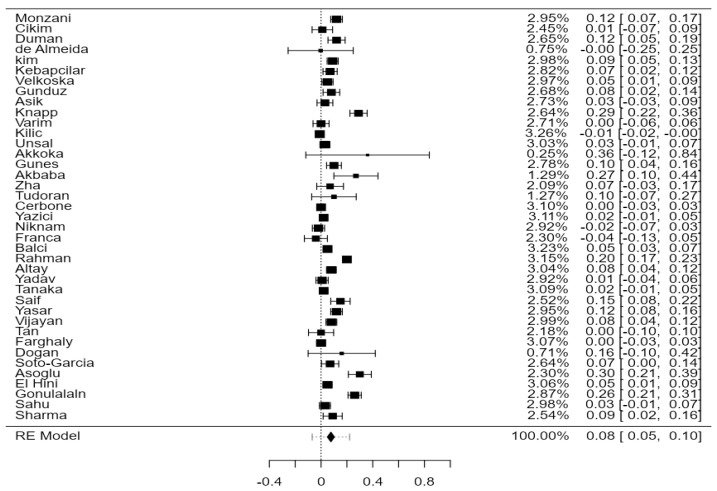
CIMT in SCH versus EU in the 39 included studies.

A total of 39 studies with 3430 subjects were analyzed, comprising 1545 SCH and 1885 EU. The observed mean difference ranged from −0.04 to 0.36, with most studies showing a positive association between SCH and the thickening of the carotid wall (79%). The estimated average mean difference between SCH and EU was 0.08 [0.05–0.10], statistically significant, *p* < 0.01 (Figure 2). The heterogeneity of the studies was high (I^2^ = 93.82%).

**Figure 3:**
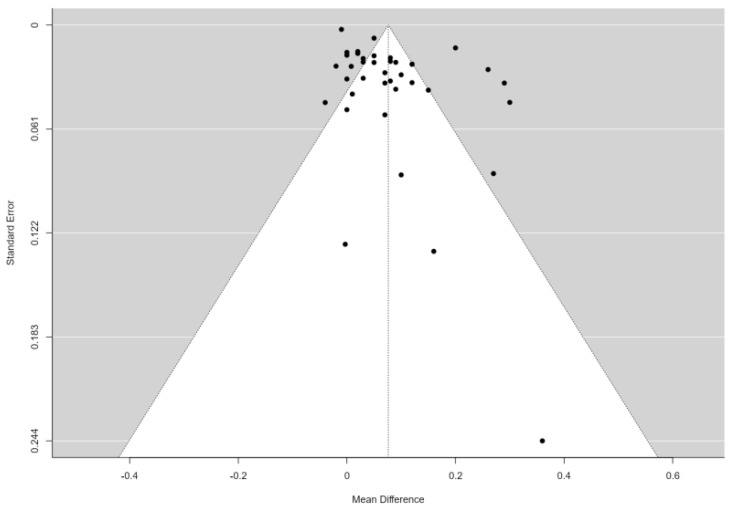
Funnel plot for the 39 studies included in the analysis.

The publication bias was not statistically significant; neither the rank correlation nor the regression test indicated any funnel plot asymmetry (*p* = 0.96 and *p* = 0.060, respectively), with most cases distributed at the top of the funnel plot (Figure 3).

By using the sex ratio, age ratio, BMI ratio, LDL ratio, HDL ratio, and triglycerides ratio as moderators, the statistical significance of the study was not modified.

We conducted a sensitivity analysis by excluding single studies and performing additional analysis for each study excluded. None of the excluded studies had a significant effect on the overall results of the meta-analysis. To ensure the accuracy of the results, we excluded studies that did not provide information about how CIMT and/or TSH were assessed, studies that utilized outdated or less accurate ultrasound devices to measure CIMT, studies that did not specify the cut-off value for TSH, studies that reported CIMT values well below the lower limit of the normal range, studies that included pediatric populations, studies that did not report BMI values or did not provide a clear explanation of how BMI values were calculated, and studies that showed significant numerical differences between the control group and the study group. After conducting further analysis of the excluded categories, it was observed that there was no significant impact on the overall statistical significance of the meta-analysis. The level of heterogeneity remained highly significant, and the moderators, including sex, age, LDL, HDL, triglycerides, and BMI ratio, did not alter the statistical significance of the results.

**Figure 4:**
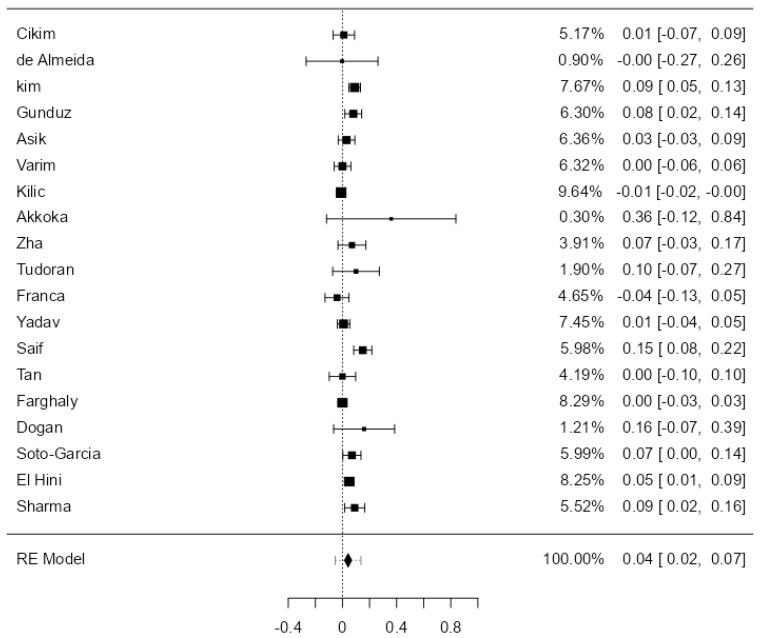
CIMT in SCH versus EU in the 19 analyzed studies.

After excluding all the abovementioned study categories once, except for the studies on pediatric populations, a total of 19 studies were analyzed. The observed mean difference ranged from −0.04 to 0.36, with most studies showing a positive association between SCH and the thickening of the carotid wall (68%). The estimated average mean difference between SCH and Eu was 0.04 [0.02–0.07], statistically significant, *p* = 0.03 (Figure 4). The heterogeneity of the studies was most likely substantial (I^2^ = 77.7%). 

After performing the sensitivity analysis, no significant statistical changes were observed in the overall effect, indicating that the meta-analysis was stable.

**Figure 5:**
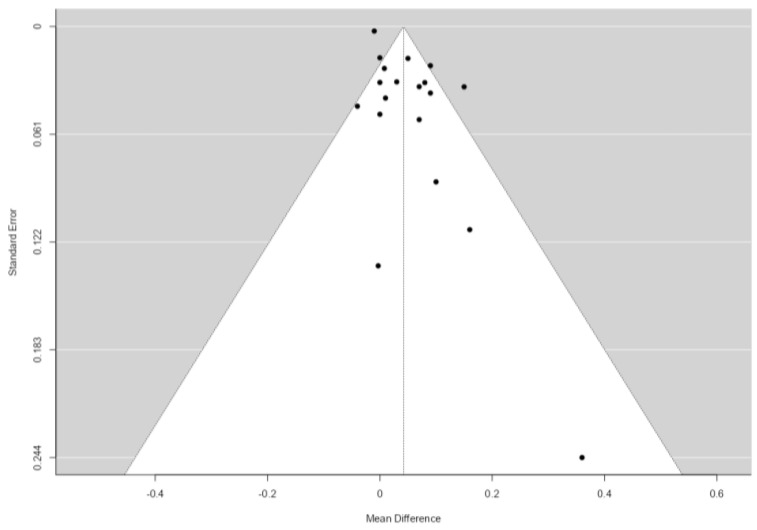
Funnel plot for the 19 analyzed studies.

For the 19 studies that remained after applying the abovementioned exclusion criteria, the publication bias was not statistically significant; neither the rank correlation nor the regression test indicated any funnel plot asymmetry (*p* = 0.89 and *p* = 0.10, respectively), with most cases distributed at the top of the funnel plot (Figure 5).

**Figure 6:**
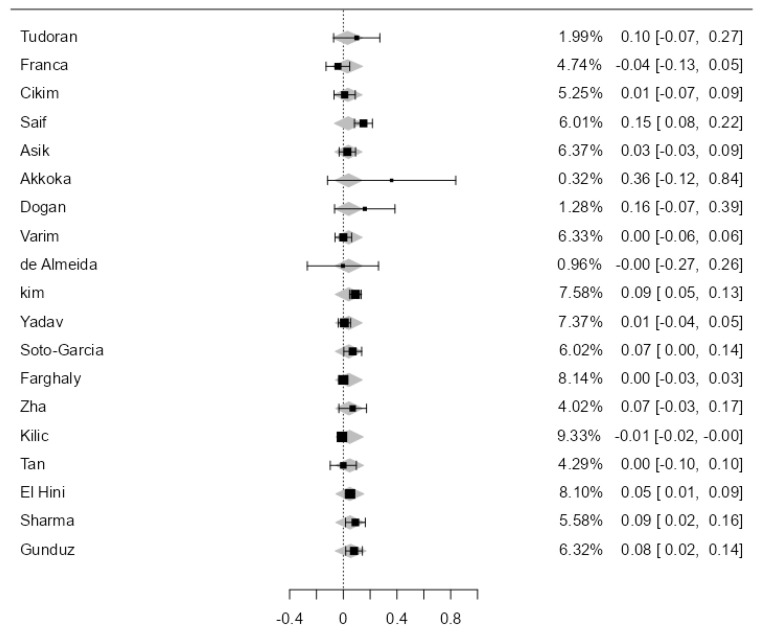
CIMT in SCH versus EU in the 19 included studies, using age ratio as a moderator.

By using the age ratio as a moderator for the abovementioned 19 studies, the statistical significance of the meta-analysis was changed, *p* = 0.07 [0.01–0.06]. The heterogeneity of the studies was most likely substantial (I^2^ = 76.7%). Advanced age was a confounding factor in the studied correlation (Figure 6).

**Figure 7:**
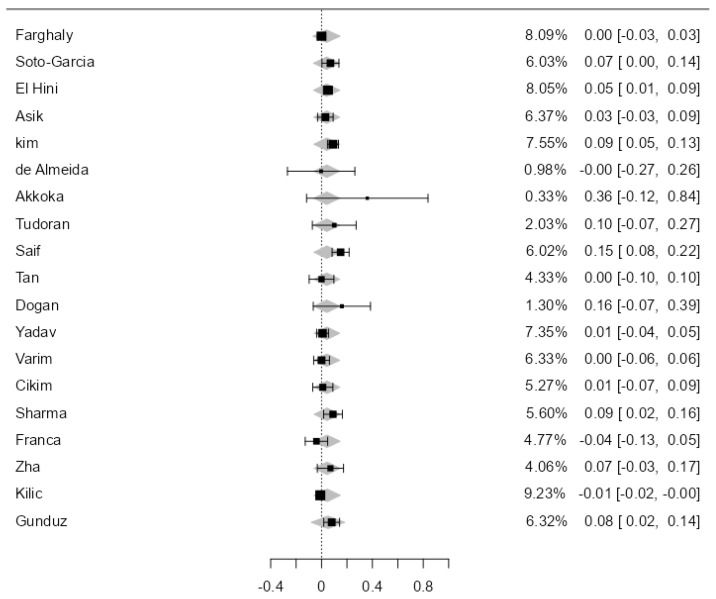
CIMT in SCH versus EU in the 19 included studies, using sex ratio as a moderator.

By also using sex ratio as a moderator for the abovementioned 19 studies, the statistical significance of the meta-analysis was changed, *p* = 0.06 [0.01–0.07]. The heterogeneity of the studies was most likely substantial (I^2^ = 74.03%). The SCH–CIMT correlation was influenced by the sex of the subjects, female sex being a confounding factor in this regard (Figure 7).

**Figure 8:**
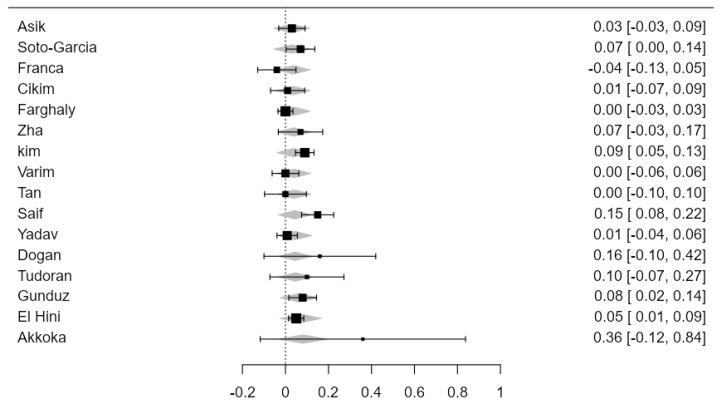
CIMT in SCH versus EU in the 16 included studies, using cholesterol ratio as a moderator.

Regarding the lipidic profile, using cholesterol ratio as a moderator for the abovementioned 19 studies that exposed this metabolic parameter (16 studies), the statistical significance of the meta-analysis was changed, *p* = 0.08 [0.01–0.06]. The heterogeneity of the studies was reduced (I^2^ = 54.43%). The SCH–CIMT correlation was influenced by high cholesterol levels (Figure 8).

Using BMI ratio, LDL ratio, HDL ratio, and triglycerides ratio as moderators, the statistical significance of the study was not modified and the heterogeneity of the studies was most likely substantial.

By filtering the studies depending on the cut-off value for TSH, with a threshold of 4.2, the statistical significance of the study was not modified, *p* = 0.04 [0–0.08], with substantial heterogeneity (I^2^ = 68.85%).

## 4. Discussion

This meta-analysis indicates a significant positive correlation between SCH and in-creased CIMT, with some limitations. Additionally, female sex, advanced age, and high cholesterol levels significantly influenced this correlation.

A previous meta-analysis on this topic, conducted by Gao et al. approximately 10 years ago within eight studies, obtained similar results [61]. Yao et al., in a meta-analysis that included 27 case-control studies in which they analyzed potential non-invasive markers for cardiovascular risk in people with subclinical hypothyroidism, found a significantly positive association between SCH and the thickening of the arterial wall, with increased risk of cardiovascular pathologies [62]. CIMT may also be a predictor for the risk of ischemic stroke. Sahoo et al. measured CIMT at the level of the common carotid artery among patients with ischemic stroke and found them to have an average of 0.798 mm CIMT, while in the control group the mean CIMT was 0.6 mm, a statistically significant difference [63]. CIMT at the level of the common carotid artery is a factor that helps predict cardiovascular risks, and the evaluation of this parameter at the level of the internal carotid artery improves its classification [64]. A recently conducted joint study of 9020 U.S. adults, by Inoue et al., found that cardiovascular disease mediated the association between subclinical hypothyroidism and all-cause mortality, especially among women and the elderly [65]. According to a study conducted by Vaya et al., patients with SCH show an increased risk of cardiovascular disease when compared to those with EU. This is characterized by higher levels of plasma viscosity, fibrinogen, homocysteine, and erythrocyte distribution [66]. In postmenopausal women, SCH has been associated with increased levels of inflammatory markers such as CRP, homocysteine, uric acid, and TNFα [67]. 

From a pathophysiological standpoint, the mechanisms of these changes are derived from the role of the thyroid hormones on metabolic parameters, including lipoprotein metabolism [68]. With an increase in TSH levels, there was an increase in cholesterol, triglycerides, and LDL-c, and a decrease in HDL-c levels; this association has a linear character [69]. People with subclinical hypothyroidism have a higher increased risk compared to euthyroid patients of developing hypercholesterolemia, increased levels of LDL-c and CRP, and elevated diastolic blood pressure [70]. The major cardiovascular risk factors are diabetes, central obesity, dyslipidemia, elevated LDL-c levels, and high blood pressure [71], which entails an increased risk of atheromatosis and myocardial ischemia. However, the etiology of hypothyroidism does not seem to influence these cardio-vascular metabolic parameters. For example, antithyroid peroxidase antibodies have not been positively associated with cardiovascular risk in patients with subclinical hypothyroidism [72]. 

Subclinical hypothyroidism is common in medical practice, and its diagnosis should consider demographics relative to the TSH reference range in the healthy population. According to the literature, in a considerable proportion of patients, subclinical hypothyroidism can be physiologically reversible, without any medication in this regard, but there are also persistent, progressive forms, mainly against the background of chronic autoimmune thyroiditis. Once subclinical hypothyroidism is detected, the patient requires periodic medical evaluations to allow risk stratification [73,74].

Studies that examined the evolution of pre-existing cardiac pathology in people with newly diagnosed SCH found a worsening of the cardiac pathology prognosis compared to that of euthyroid patients, during the follow-up period of less than 5 years observed in patients with SCH, including the need for ventricular stimulation and heart transplantation, or even death [74,75,76]. Corona et al. suggest that subclinical hypothyroidism affects cardiovascular risk factors, but its effects are mediated by the pre-existence/coexistence of these risk factors, instead of terminating a specific pathophysiological mechanism [77].

Regarding effective treatment to minimize cardiovascular consequences, meta-analyses have revealed the positive effect of levothyroxine administration, which led to a decrease in CIMT [78,79]. In a meta-analysis of 119 clinical trials [80], it was seen that following a decrease in CIMT progression, cardiovascular risks decreased. In contrast, in a randomized study of 185 elderly patients, administration of levothyroxine treatment for one year did not reduce the progression of CIMT compared to the placebo group [81].

According to the last ETA guideline issued 10 years ago for the treatment of subclin-ical hypothyroidism, treatment suggestions take into account the patient’s age and serum TSH, with a reference age of 70 years and a TSH level of 10 mUI/mL. LT4 therapy is considered per primam only if TSH has values greater than or equal to 10 mUI/mL. For people under 70 years of age, it is recommended that LT4 be administered, and in people over 70 years of age, LT4 is recommended only if there are clear symptoms of hypothyroidism or increased cardiovascular risk [82]. These indications are derived from studies that demonstrated the effectiveness of LT4 treatment in subclinical hypothyroidism in people under 70 years of age, whereas no decrease in ischemic cardiac events was observed in persons above this age limit [83]. One explanation for these results could be the etiology of subclinical hypothyroidism, which in young people has a more frequent autoimmune etiology, while in the elderly it was found that it can also be a change without pathological significance but was rather physiological, as revealed by the study of Surks and Hollowell, who concluded that TSH levels increase progressively, physiologically, with age [84].

The applicability of this research in clinical medical practice could lie in its potential for non-invasive screening to predict cardiovascular risk in patients with subclinical hypothyroidism, as well as the prompt analysis of optimal therapy. At the same time, the positive association between SCH and atheromatosis could be a potential indication for thyroid function testing in patients with cardio-vascular pathology.

The limitations of this meta-analysis were: the different cut-off values of TSH between studies and the lack of uniformity in the evaluation of the THS value; some studies did not specify the TSH assessment method; the lack of metabolic profile analysis in some studies; the low number of persons in the analyzed studies; the different number of persons in the study group versus the control group in some studies; studies limited to certain age groups; the narrow geographical distribution of the studies- out of the 39 studies included in the meta-analysis, the majority (19 original studies) took place in Turkey, leading to an absence of evidence from Western developed countries.

The small number of randomized clinical trials and cohort studies on this topic, often considered more adequate at establishing cause–effect relationships compared to case-control studies, represents another study limitation.

Also as a limitation, the absence in the included studies of a normal value, threshold of CIMT, and additional data regarding classic cardiovascular risk factors, such as menopause, smoking, alcohol consumption, blood pressure, diabetes mellitus, and diet, should be considered as potential confounding factors. In addition, in the studies on this topic, no data were found to attest to the pathological role of subclinical hypothyroidism per se in terms of clinical cardiovascular complications due to subclinical hypothyroidism.

As another limitation, the significant heterogeneity among studies was very important. Even if we could not identify all the studies that were the sources of this heterogeneity, the stability of the outcomes was confirmed after adjusting for potential publication bias.

## 5. Conclusions

These research findings suggest, with some limitations, a statistically significant positive association between SCH and increased CIMT. There are necessary large-scale, prospective studies to be conducted on substantial populations, taking into account traditional cardiovascular risk factors and incorporating long-term follow-up for accurate risk stratification and optimal therapeutic indication.

## Figures and Tables

**Table 1:** Studies included in meta-analysis.

Author	Year	Country	TSH Cut off Value (mUI/mL)	Participants (SCH/EU)	Age (SCH/EU)	NOS
Monzani [20]	2004	Italy	>3.6	45/32	37 ± 1/35 ± 1	8
Cikim [21]	2004	Turkey	>4.20	25/23	32.2 ±9.6/35.8 ± 7.9	7
Duman [22]	2007	Turkey	>4.2	40/20	37 ± 12.6/36.7 ± 12.2	7
de Almeida [23]	2007	Brazil	>4	30/27	43 ± 9.7/43.1 ± 8.3	8
Kim [24]	2009	Korea	>5.5	36/32	36 ± 6.2/36.1 ± 5.4	7
Kebapcilar [25]	2010	Turkey	>5	38/19	49.7 ± 10/49.9 ± 8.1	8
Velkoska [26]	2011	Macedonia	>4.2	67/30	42.4 ± 16.2/43.6 ± 12.8	7
Gunduz [27]	2012	Turkey	>4	16/20	40.8 ± 11.8/32.8 ± 5.7	7
Asik [28]	2013	Turkey	>5.49	33/32	38.1 ± 15/39.4 ± 9.7	8
Knapp [29]	2013	Poland	–	40/15	34.8 ± 4.1/31.6 ± 9.3	4
Varim [30]	2013	Turkey	>4.5	50/50	29.5 ± 8.9/29.8 ± 7.6	7
Kilic [31]	2013	Turkey	>4.2	32/29	41.5 ± 12/38.1 ± 11.4	5
Unsal [32]	2014	Turkey	>4.2	56/46	41.3 ± 14.4/36 ± 10.5	7
Akkoca [33]	2014	Turkey	>4.2	20/20	34.4 ± 1.4/35.2 ± 2.2	7
Gunes [34]	2014	Turkey	>4.2	39/29	40.4 ± 15.3/41 ± 13.8	8
Akbaba [35]	2015	Turkey	>4	51/43	36.9 ± 10/34.9 ± 8.4	8
Zha [36]	2015	China	>4.5	10/10	53.2 ± 5.4/52 ± 5.7	7
Tudoran [37]	2015	Romania	>4.2	15/15	36.7 ± 5.2/42.1 ± 6.8	4
Yazici [38]	2015	Turkey	>4	43/30	35.2 ± 1/34.5 ± 8.2	8
Niknam [39]	2016	Iran	>4	25/25	35.9 ± 7.6/37.5 ± 7.3	8
Fraca [40]	2016	Brazil	>4.5	16/15	39.6 ± 10.1/45 ± 7.4	5
Cerbone [41]	2016	Italy	>4.2	39/39	9.1 ± 3.5/9.4 ± 3.6	8
Isik-Balci [42]	2016	Turkey	NA	53/31	9.2 ± 4.2/7.1 ± 5.1	4
Rahman [43]	2016	Bangladesh	>5	26/30	30 ± 7.4/32 ± 8.7	8
Altay [44]	2017	Turkey	NA	35/30	34.4 ± 10.3/32.5 ± 7.5	5
Yadav [45]	2017	India	>7.5	27/20	10.9 ± 2.3/10.8 ± 2.4	8
Tanaka [46]	2018	Japan	>4.5	55/674	60.1 ± 7/56.1 ± 9.4	5
Saif [47]	2018	Egypt	>4.8	30/40	34 ± 8/36 ± 4.8	8
Yasar [48]	2018	Turkey	>5.6	160/86	39.5 ± 14.8/40.4 ± 10.0	7
Vijayan [49]	2018	India	>4.2	30/30	33.9 ± 10.6/37.9 ± 9.7	8
Tan [50]	2019	Turkey	>4.94	40/40	32 ± 27/28 ± 19	8
Farghaly [51]	2019	Egypt	>4	32/32	13.6 ± 2.4/13.2 ± 2.1	8
Dogan [52]	2019	Turkey	>4.2	50/37	35.3 ± 9.5/35.6 ± 1.9	6
Soto-Garcia [53]	2020	Mexic	>4	18/18	37 ± 12.9/36.8 ± 12.7	8
Asoglu [54]	2021	Turkey	>4.2	80/43	44.0 ± 13.1/46.7 ± 8.3	7
El Hini [55]	2021	Egypt	>4.5	36/36	40.2 ± 8.6/35.6 ± 9.9	6
Gonulalaln [56]	2021	Turkey	>4	30/52	35 ± 13.6/38.7 ± 10.4	7
Sahu [57]	2022	India	>5	42/50	10.3 ± 3.7/10.1 ± 3.1	7
Sharma [58]	2022	India	>4.2	35/35	46.2 ± 10/41.1 ± 11.1	6

**Table 2:** CIMT and TSH assessment in analyzed studies.

Authors	Year	CIMT Assay	TSH Assay
Monzani [20]	2004	High-resolution ultrasonography using multiple equipment types and 7.5 MHz linear transducer—multiple measurements of both carotid arteries and internal carotid arteries	Ultrasensitive immunoradiometric assay (IRMA) method
Cikim [21]	2004	High-resolution ultrasound imaging (Vingmed System Five, 10 mH linear probe)—both common carotid arteries; three measurements from each subject	Autoanalyzer Roche/Hitachi Modular System—method not specified
Duman [22]	2007	High-resolution ultrasonography with a 7.5 MHz linear array transducer using a vascular ultrasound system (ATL-3500-HDI; Philips Medical Systems, Andover, MA, USA)—multiple measurements of both common carotid arteries	Roche/Hitachi modular analytics SWA—immunoassay
de Almeida [23]	2007	High-resolution ultrasound with Acusson Aspem Advanced and 10 MHz linear transducer—multiple measurements of both common carotid arteries and bifurcation	Imunometric measurement (IMMULITE DPC^®^)
Kim [24]	2009	High-resolution ultrasonographic system (Prosound α10) with 10.0 MHz linear transducer—multiple measurements in the mid and distal portion of the common carotid arteries	Chemiluminescent immunometric assay
Kebapcilar [25]	2010	High-definition ultrasonography (Philips HDI 5000) with L12-5 linear wide-band probe—two measurements, one proximal and one distal for each common carotid artery	Chemiluminescent immunometric assay (Immulite 2000)
Velkoska [26]	2011	Ultrasound system HP Agilent S4500 with 7.5–10.0 MHz linear transducer—two measurements of the right carotid artery	Immulite 2000 chemiluminescent analyzer
Gunduz [27]	2012	Gray-scale high-resolution color Doppler ultrasound (Prosound SSD—3500 SV ALOKA)—one measurement for each common carotid artery	Immulite 2000 chemiluminescent analyzer
Asik [28]	2013	Echocardiography machine VIVID 3 equipped with linear-array imaging probe—one measurement 10 mm proximal to the right carotid artery bifurcation	Chemiluminometric method (ADVIA Centaur analyzer
Knapp [29]	2013	Ultrasound imaging (Philips iE33) with 1–5 MHz sector transducer and 3–11 MHz linear-array high-resolution transducer—two measurements for each common carotid artery	Method not specified
Varim [30]	2013	Ultrasound with Siemens Sonoline Ultrasound using a 10 MHz linear probe—three measurements for each common carotid artery	Method not specified
Kilic [31]	2013	Ultrasound imaging (Vivid 7 dimensions) with 12 MHz linear array transducer—two measurements for each common carotid artery	Immunochemiluminescence method (Cobalt 6000 analyzer)
Unsal [32]	2014	High-resolution ultrasonography (Hitachi EUB 7000 HV) with 13 MHz probe—three measurements for each common carotid artery	Chemiluminescence assay (Advia Centaur) and specific electrochemiluminescence immunoassay (Elecsys 2010 Cobas)
Akkoca [33]	2014	Gray-scale, high-resolution color Doppler ultrasound (Siemens) with 13 MHz linear transducer—one measurement for each carotid artery	Chemiluminescence method (Immulite 2000)
Gunes [34]	2014	Ultrasound imaging (VIVID 3 machine) with 2.5 MHz linear-array probe—three measurements approximately 10 mm proximal of the carotid bifurcation for the right common carotid artery	Electro-chemiluminescence immunoassay “ECLIA” (Roche Cobas E601 analyzer)
Akbaba [35]	2015	High-resolution B-mode ultrasound (Loqic 3 device) with 11 MHz linear array transducer—three measurements for each common carotid artery	Chemiluminescence micro-particle immunoassay (Abbot Architect 2000)
Zha [36]	2015	Color ultrasound (Toshiba Aplio 500) with 9 Mhz linear-array transducer—three measurements for each common carotid artery	Chemiluminescence procedure (Roche Cobas E610)
Tudoran [37]	2015	Echocardiography device (Aloka CV Prosound SSD-4000 SV) with 10 MHz linear transducer—five measurements for each carotid artery beginning from carotid bulb dilation	Method not specified
Yazici [38]	2015	Ultrasound imaging (GE Vingmed) with 10 MHz broadband linear probe—number of measurements and sites not specified	Method not specified
Nikna [39]	2015	Sonogram B-mode imaging—number of measurements and sites not specified	Method not specified
França [40]	2016	Ultrasound imaging with 7.5 MHz multifrequency linear array probe (device not specified)—three measurements of the common carotid artery	Electrochemiluminescence immunoassay (Roche Diagnostics kits)
Cerbone [41]	2016	Ultrasound imaging (GE Vivid I) with 7.0 MHz—multiple measurements above the carotid sinus for both common carotid arteries	Chemiluminescence method (Immulite 2000)
Isik-Balci [42]	2016	Ultrasound imaging (Logiq E9 ultrasound) with a 6–15 MHz linear array probe—three measurements 20 mm proximal to the carotid bifurcation	Electrochemiluminescence (Roche Cobas 6000)
Rahman [43]	2016	Ultrasound imaging (DC-7 scanner) with 7.5–10 MHz linear transducer—one measurement 1.5 cm superior to the carotid bifurcation for each carotid artery	Immunoradiometric assay
Altay [44]	2017	Ultrasound imaging (General Electric Logic 5 Pro) with 12 MHz—five measurements for each common carotid artery, 1 cm distal to the main carotid artery bulb	Method not specified
Yadav [45]	2017	B-mode ultrasound imaging (Siemens) with 10 MHz linear transducer—number of measurements and sites not specified	Chemiluminescence immunoassay (IMMULITE 1000)
Tanaka [46]	2018	High-resolution B-mode ultrasonography (UF-4300R) with 7.5 MHz linear array probe—unspecified number of measurements for both common carotid arteries 20 mm proximal to the carotid bulb	CLIA immunoassay
Saif [47]	2018	High-resolution color-codded Doppler ultrasonography (ALT HDI) with 12 MHz linear array probe—four measurements for both common carotid arteries	Method not specified
Yasar [48]	2018	Ultrasound imaging (Toshiba Aplio 300) with 9–13 MHz linear probe—unspecified number of measurements 2 cm proximal to the carotid bulb	Chemiluminescent method (Immulite 2000)
Vijayan [49]	2018	Ultrasound imaging (Mindray DC-8) with 7 MHz linear transducer—unspecified number of measurements 10 mm proximal to the right common carotid artery	Chemiluminescent immunometric assay
Tan [50]	2019	B-mode ultrasonography with 7.5–13.5 MHz multifrequency linear array probe—three measurements for each common carotid artery	Electrochemiluminescence method (Abbot Aeroset kit)
Farghaly [51]	2019	Color duplex flow imaging (Acuson 128 XP)—three measurements at 1–2 cm proximal to the carotid bulb for each common carotid artery	Ultrasensitive immunometric assay (Immulite 2000 Third Generation)
Dogan [52]	2019	Ultrasonography (Aloka Prosound SSD 5000) with 7.5 MHz linear probe—unspecified number of measurements 10 mm proximal to the bifurcation for each common carotid artery	Electrochemiluminescence assay
Soto-Garcia [53]	2020	B-mode ultrasonography with 7.5–13.5 MHz multifrequency linear array probe—three measurements for each common carotid artery	Method not specified
Asoglu [54]	2021	Unspecified equipment and number of measurements 1–2 cm proximal to the carotid artery bifurcation	Chemiluminescence methods
El Hini [55]	2021	Method not specified	Enzyme-linked fluorescence immunoassay (BioMerieux Mini Vidas)
Gonulalaln [56]	2021	B-mode ultrasonography (LOGIQ P5)—three measurements 1 cm proximal to the bifurcation for each common carotid artery	Method not specified
Sahu [57]	2022	Color duplex flow imaging (Samsung HS 70 A) with 7 MHz probe—unspecified number of measurements for both common carotid arteries	Electrochemiluminescence assay (Roche Cobas 411)
Sharma [58]	2022	Unspecified equipment and number of measurements 1 cm proximal to the carotid artery bifurcation	Method not specified

**Table 3:** Carotid intima-media thickness (CIMT) and body mass index (BMI) in subclinical hypothyroidism (SCH) versus euthyroidism (EU) in the analyzed studies.

Authors	SCH_BMI	EU_BMI	SCH_CIMT	EU_CIMT
Monzani [20]	24.7 ± 3.5	24.2 ± 3.7	0.75 ± 0.13	0.63 ± 0.07
Cikim [21]	26.03 ± 6.21	27.04 ± 4.95	0.55 ± 0.14	0.54 ± 0.14
Duman [22]	25.1 ± 4.3	24.7 ± 2.5	0.66 ± 0.16	0.54 ± 0.10
de Almeida [23]	27.3 ± 4.6	25.41 ± 4.38	0.57 ± 0.70	0.57 ± 0.68
Kim [24]	23.1 ± 2.8	23.3 ± 3.1	0.66 ± 0.10	0.57 ± 0.08
Kebapcilar [25]	28.58 ± 5.81	28.45 ± 5.25	0.64 ± 0.13	0.57 ± 0.08
Velkoska [26]	27.8 ± 5.6	25.4 ± 5.1	0.61 ± 0.1	056 ± 0.1
Gunduz [27]	26.72 ± 2.32	24.34 ± 2.43	0.61 ± 0.11	0.53 ± 0.08
Asik [28]	30.37 ± 7.67	27.79 ± 3.64	0.54 ± 0.14	0.51 ± 0.11
Knapp [29]	24.43 ± 4.3	21.8 ± 1.48	0.61 ± 0.14	0.32 ± 0.1
Varim [30]	25.7 ± 4	25.66 ± 4.24	0.4 ± 0.2	0.4 ± 0.1
Kilic [31]	28.6 ± 5.9	24.9 ± 6.5	0.05 ± 0.01	0.06 ± 0.01
Unsal [32]	-	-	0.53 ± 0.11	0.5 ± 0.86
Akkoca [33]	28.42 ± 1.86	27.97 ± 4.15	0.74 ± 0.8	0.38 ± 0.74
Gunes [34]	28.79 ± 6.6	27.46 ± 5.35	0.65 ± 0.13	0.55 ± 0.11
Akbaba [35]	26.1 ± 5.5	25.7 ± 4.2	0.74 ± 0.3	0.47 ± 0.5
Zha [36]	24.4 ± 1.8	24 ± 1.6	0.82 ± 0.14	0.75 ± 0.09
Tudoran [37]	26.24 ± 2.7	27.5 ± 6.71	0.72 ± 0.14	0.62 ± 0.31
Yazici [38]	25.1 ± 5.6	25.0 ± 4.1	0.50 ± 0.09	0.48 ± 0.04
Niknam [39]	26 ± 2	25.82 ± 2	0.56 ± 0.09	0.58 ± 0.08
França [40]	26.5 ± 4.4	24.6 ± 2.98	0.62 ± 0.11	0.66 ± 0.14
Cerbone [41]	-	-	0.44 ± 0.08	0.44 ± 0.06
Isik-Balci [42]	17.56 ± 3.61	17.56 ± 2.47	0.5 ± 0.09	0.43 ± 0.03
Rahman [43]	25.6 ± 4.7	-	0.08 ± 0.05	0.6 ± 0.05
Altay [44]	27.6 ± 5.9	23.7 ± 3.9	0.63 ± 0.10	0.55 ± 0.05
Yadav [45]	17.79 ± 4.11	15.99 ± 1.69	0.48 ± 0.07	0.47 ± 0.08
Tanaka [46]	22.1 ± 2.7	22.1 ± 3.1	0.59 ± 0.12	0.57 ± 0.1
Saif [47]	26 ± 3.6	24 ± 2.3	0.6 ± 0.2	0.45 ± 0.07
Yasar [48]	30.22 ± 5.71	29.6 ± 6.12	0.55 ± 0.13	0.43 ± 0.19
Vijayan [49]	24.66 ± 4.13	22.86 ± 3.01	0.55 ± 0.10	0.47 ± 0.06
Tan [50]	23.67 ± 5.37	21.39 ± 3.52	0.5 ± 0.27	0.5 ± 0.16
Farghaly [51]	-	-	0.44 ± 0.08	0.44 ± 0.06
Dogan [52]	25.2 ± 3.8	31.4 ± 44.9	0.59 ± 0.12	0.43 ± 0.8
Soto-Garcia [53]	26.8 ± 4.7	29.6 ± 3.6	0.49 ± 0.12	0.42 ± 0.07
Asoglu [54]	26.5 ± 2.4	26.2 ± 2.4	0.8 ± 0.3	0.5 ± 0.2
El Hini [55]	26.7 ± 2	23.3 ± 0.84	0.56 ± 0.09	0.51 ± 0.06
Gonulalaln [56]	29.87 ± 5.09	29.12 ± 5.83	0.61 ± 0.11	0.35 ± 0.12
Sahu [57]	20.39 ± 2.51	18.81 ± 3.13	0.52 ± 0.12	0.49 ± 0.08
Sharma [58]	23.76 ± 1.77	23.12 ± 1.73	0.68 ± 0.14	0.59 ± 0.17

**Table 4:** CIMT in SCH vs. EU depending on TSH cut-off values.

Author	Year	TSH Cut off Value (mUI/mL)	SCH_CIMT	EU_CIMT
Monzani [20]	2004	>3.6	0.75 ± 0.13	0.63 ± 0.07
Cikim [21]	2004	>4.20	0.55 ± 0.14	0.54 ± 0.14
Duman [22]	2007	>4.2	0.66 ± 0.16	0.54 ± 0.10
de Almeida [23]	2007	>4	0.57 ± 0.70	0.57 ± 0.68
Kim [24]	2009	>5.5	0.66 ± 0.10	0.57 ± 0.08
Kebapcilar [25]	2010	>5	0.64 ± 0.13	0.57 ± 0.08
Velkoska [26]	2011	>4.2	0.61 ± 0.1	056 ± 0.1
Gunduz [27]	2012	>4	0.61 ± 0.11	0.53 ± 0.08
Asik [28]	2013	>5.49	0.54 ± 0.14	0.51 ± 0.11
Knapp [29]	2013	–	0.61 ± 0.14	0.32 ± 0.1
Varim [30]	2013	>4.5	0.4 ± 0.2	0.4 ± 0.1
Kilic [31]	2013	>4.2	0.05 ± 0.01	0.06 ± 0.01
Unsal [32]	2014	>4.2	0.53 ± 0.11	0.5 ± 0.86
Akkoca [33]	2014	>4.2	0.74 ± 0.8	0.38 ± 0.74
Gunes [34]	2014	>4.2	0.65 ± 0.13	0.55 ± 0.11
Akbaba [35]	2015	>4	0.74 ± 0.3	0.47 ± 0.5
Zha [36]	2015	>4.5	0.82 ± 0.14	0.75 ± 0.09
Tudoran [37]	2015	>4.2	0.72 ± 0.14	0.62 ± 0.31
Yazici [38]	2015	>4	0.50 ± 0.09	0.48 ± 0.04
Niknam [39]	2016	>4	0.56 ± 0.09	0.58 ± 0.08
Franca [40]	2016	>4.5	0.62 ± 0.11	0.66 ± 0.14
Cerbone [41]	2016	>4.2	0.44 ± 0.08	0.44 ± 0.06
Isik-Balci [42]	2016	NA	0.5 ± 0.09	0.43 ± 0.03
Rahman [43]	2016	>5	0.08 ± 0.05	0.6 ± 0.05
Altay [44]	2017	NA	0.63 ± 0.10	0.55 ± 0.05
Yadav [45]	2017	>7.5	0.48 ± 0.07	0.47 ± 0.08
Tanaka [46]	2018	>4.5	0.59 ± 0.12	0.57 ± 0.1
Saif [47]	2018	>4.8	0.6 ± 0.2	0.45 ± 0.07
Yasar [48]	2018	>5.6	0.55 ± 0.13	0.43 ± 0.19
Vijayan [49]	2018	>4.2	0.55 ± 0.10	0.47 ± 0.06
Tan [50]	2019	>4.94	0.5 ± 0.27	0.5 ± 0.16
Farghaly [51]	2019	>4	0.44 ± 0.08	0.44 ± 0.06
Dogan [52]	2019	>4.2	0.59 ± 0.12	0.43 ± 0.8
Soto-Garcia [53]	2020	>4	0.49 ± 0.12	0.42 ± 0.07
Asoglu [54]	2021	>4.2	0.8 ± 0.3	0.5 ± 0.2
El Hini [55]	2021	>4.5	0.56 ± 0.09	0.51 ± 0.06
Gonulalaln [56]	2021	>4	0.61 ± 0.11	0.35 ± 0.12
Sahu [57]	2022	>5	0.52 ± 0.12	0.49 ± 0.08
Sharma [58]	2022	>4.2	0.68 ± 0.14	0.59 ± 0.17

## Data Availability

The original data presented in the study are openly available in PubMed, MedLine, Scopus, and Web of Science databases.
